# What Conditions Make Proton Beam Therapy Financially Viable in Western Canada?

**DOI:** 10.7759/cureus.3644

**Published:** 2018-11-27

**Authors:** Wendy L Smith, Craig D Smith, S Patel, David D Eisenstat, Sarah Quirk, Marc Mackenzie, Ivo A Olivotto

**Affiliations:** 1 Medical Physics, University of Calgary, Calgary, CAN; 2 Miscellaneous, University of Calgary, Calgary, CAN; 3 Radiation Oncology, University of Alberta, Alberta, CAN; 4 Pediatric Oncology, University of Alberta, Alberta, CAN; 5 Medical Physics, University of Alberta, Alberta, CAN; 6 Oncology, University of Calgary/Tom Baker Cancer Center, Calgary, CAN

**Keywords:** proton therapy, health economics, proton beam therapy, radiation therapy

## Abstract

Background

Proton beam therapy (PBT) is available in many western and Asian countries, but there is no clinical, gantry-based PBT facility in Canada.

Methods

A cost analysis was conducted from the Alberta Ministry of Health perspective with a 15-year horizon. Estimated costs were: PBT unit, facility development as part of an ongoing capital project, electricity, maintenance contract, and staffing. Revenues were: savings from stopping USA referrals, avoiding the costs of standard radiation therapy (RT) for Albertans receiving PBT instead, and cost-recovery charges for out-of-province patients.

Results

The Ministry of Health funded 15 Albertans for PBT in the USA in the 2014/15 fiscal year (mean CAD$ 237,348/patient). A single-vault, compact PBT unit operating 10 hours/day could treat 250 patients annually. A 100 Albertans, with accepted indications, such as the curative-intent treatment of chordomas, ocular melanomas, and selected pediatric cancers, would likely benefit annually from PBT’s improved conformality and/or reduced integral dose compared to RT. The estimated capital cost was $40 million for a single beamline built within an ongoing capital project. Operating costs were $4.8 million/year at capacity. With 50% capacity reserved for non-Albertans at a cost recovery of $45,000/patient, a Western Canadian PBT facility would achieve net positive cash flow by year eight of clinical operations, assuming Alberta-to-USA referrals reach 21 patients/year by 2024 and increase at 3%/year thereafter. Sensitivity analysis indicates the lifetime net savings is robust to the assumptions made.

Conclusion

This business case, based on Canadian costing data and estimates, demonstrates the potential for a financially viable PBT facility in Western Canada.

## Introduction

Proton beam therapy (PBT) is a specialized form of radiation therapy (RT). The theoretical advantage of PBT is that it produces a more conformal dose distribution due to the Bragg peak, with a potentially lower risk of side-effects in normal tissues and a lower radiation-induced secondary cancer risk in long-term survivors. Considerable debate surrounds the appropriate use of PBT in cancer treatment, due to the paucity of outcome studies and randomized trial data, but most experts agree that PBT is clinically justified in select cancer patients [1-5]. PBT is available in all other G7 countries and many Asian countries, but Canada does not have a clinical, gantry-based PBT facility [6]. This report examines the conditions under which it would be financially justified to provide a clinical PBT service in Western Canada.

The group of patients for which the use of protons has been well accepted is termed ‘Category A’ in the American Society for Therapeutic Radiology and Oncology (ASTRO) model policy [1]. ‘Category A’ indications for PBT include curative-intent treatment of chordomas, ocular melanomas, and certain childhood cancers [1], consistent with other published guidelines [5,7-10]. A large number of patients with additional cancer types are treated with PBT, especially in the USA and Japan, which have 19 and 11 PBT facilities, respectively [11].

The Dutch have proposed a categorization to select patients for PBT as compared to photon RT based on a model-based, personalized approach to reduce toxicity [10]. Cost-effectiveness studies of PBT in pediatric and adult populations, using Markov or Monte Carlo modeling, have compared the increased costs of PBT to avoided costs from reduced toxicity and second malignancies. Planning studies have shown that PBT achieves favorable dose distributions compared to photon RT for select patient populations [12-17]. A recent internal analysis (S Patel) of Alberta cancer treatments showed that approximately 100 Albertans per year receive a course of RT for a condition that met Alberta Health Services (AHS) published indications to consider PBT.

Prudent health care leaders should reasonably assess the relative costs and benefits before investing in PBT, but this analysis will vary by jurisdiction and opportunity. Factors influencing a specific case include geographic location and population density, cancer incidence within the population, a public versus private funding model or reimbursement mechanism, the existence and stage of supporting infrastructure and professional expertise, facility type (freestanding or integrated within a larger RT center), and RT utilization rates and referral patterns [18-19]. PBT technology is in the midst of a revolution with compact, less expensive systems under development and being marketed [18]. Several countries with publicly funded health systems similar to Canada’s have adopted or are building regional PBT services, including Sweden, Norway, Denmark, the UK, and Israel [3,10,20].

The Alberta government has committed to building a 109,000 m^2^ ‘full service’ tertiary cancer center to open in 2023 (http://www.albertahealthservices.ca/info/page15399.aspx). A single-beam line, clinical PBT service would use about 1% of the center’s planned space. This report is a financial analysis of the conditions under which it would be fiscally prudent to include PBT into the cancer center design for patients meeting ‘Category A’ indications.

## Materials and methods

Context

In 2016, the estimated population of Alberta was 4.3 million and that of Western Canada (BC, Alberta, Saskatchewan, Manitoba, and the Yukon and Northwest Territories) was 11.1 million people [21]. It is estimated that 18,600 new cancers will be diagnosed in Alberta and 56,610 in Western Canada in 2017 [22]. Western Canada covers 2.9 million square kilometers and has 14 cancer centers with standard, photon-based radiotherapy (hereafter referred to as standard RT) departments. There are no clinical, gantry-based PBT facilities in Canada. There was a research-based proton facility in Vancouver (Tri-University Meson Facility (TRIUMF)) with a fixed beam line capable of treating a limited number of patients with small choroidal melanomas, but that facility will be unavailable from October 2017 through at least 2019.

Alberta, like other Canadian provinces, has a publicly funded health system, including the provision of 100% of the RT delivered in the province. Each province has a mechanism for patients to be funded out of the country for PBT [4-5,23]. This is a report of a cost analysis conducted from the perspective of the Alberta health system to assess whether it would be financially prudent to incorporate PBT into a new cancer center being constructed in Calgary.

Assumptions

The variables, values, and rationale for assumptions used (Table 1) were based on extensive discussions with PBT vendors, utility providers, and referring physicians. The Out-of-Country Health Services Committee in Alberta provided current and past costs for standard RT and PBT delivered out-of-country. The assumptions were reviewed by a provincial committee of experts, including representatives from radiation oncology, pediatric oncology, neurosurgery, and medical physics across Alberta. For instance, it was projected that 21 patients will travel to the USA for PBT in 2024 at an average cost of $180,000 (down from $237,248 in 2015/16) and that if a PBT facility was available in Calgary, patients with indications for PBT living in Western Canada would preferentially travel to that facility if a course of PBT could be provided for just $45,000.

**Table 1:** Key assumptions used to build the financial model PBT: proton beam therapy

Variable	Value	Rationale
Annual case volume (Year 1)	120	120 patients in Year 1, ramp up 25%/y for Years 1-3 for staff training, experience, and as referral rates increase. 202 patients in Year 4. Thereafter, 3% annual growth (conservatively set at slightly less population/incidence projections). Annual treatment capacity based on reported operations from existing compact PBT facilities adjusted for 10-hour treatment day.
Annual growth in case volume (Years 4-15)	3%	
% of treated cases from Out-of-Province	50%	The authors believe all Western Canadians deserve access within their region to PBT. Requires political will to reserve capacity for most medically justified cases, regardless of the province of origin. Quality of care must be demonstrated for providers to feel confident referring to a new center.
Discount rate	2.60%	The discount rate is used to determine the present value of future cash flows. It is calculated as a function of the cost of capital. The 2016 yield on long-term (15 year) provincial bonds was 2.6%. Long-term bond yields have not fluctuated significantly in recent years, making this a realistic discount rate value.
Cases not treated in USA in 2024	21	15 patients referred in 2015-16, expected growth from cancer incidence (3.2%/year in Alberta due to both population growth and ageing (source: Alberta Cancer Registry), expanding indications for PBT, and increased awareness of PBT among physicians, patients, and families.
Cost per case treated in USA	$180,000	A conservative estimate, anticipating some savings over last available average charges ($237,248/patient in 2015-16), because cost/case may decline with greater competition as more facilities open in the USA, equipment costs may decline and/or a ‘bulk purchase’ agreement might be negotiated or the Canadian to USA dollar exchange rate might become more favorable.
Inflation Rate	2%	Canadian inflation rates over the past decade have varied from 1.8%-2.2%.
PBT equipment purchase	$30,000,000	Based on vendor discussions. Will vary depending on the type of equipment.
Facility cost allocation	$10,000,000	Incremental costs for facility construction as part of a new cancer center build. A greenfield build would cost substantially more. Based on vendor discussions.
Capital Expenses	$40,000,000	The total is PBT equipment purchase plus facility cost allocation.
Charge for Out-of-Province patients	$45,000	There is a precedent for one province to charge another for health care services provided to patients from that province. An existing Interprovincial Agreement Committee sets the fee per service used between provinces. Services not covered within the Interprovincial Agreement are charged a cost-recovery fee by the service-providing province. For instance, Saskatchewan pays Alberta $10,000 per course of prostate brachytherapy. $45,000/case will recover the initial capital investment in a reasonable period and should be substantially lower than current American PBT fees to attract sufficient out-of-province patients.
Annual increase in cases treated in the USA if no PBT in Alberta	3%	Conservatively set at slightly less than population growth and general cancer incidence projections.
Operating expenses (Years 1-3)	$3,739,739	See Table 3. Personnel costs are based on current AHS salary rates for these positions. The allocation for electricity ($200,000 per year, growing with inflation) was estimated from the cost of 0.85 GigaWatt-hours per year (the high end of electrical power consumption estimates from three PBT unit vendors) and the commercial cost of electrical power based on consultation with sources at the City of Calgary and ENMAX Energy Corporation. The lower operating cost for the initial three years is because the facility is not expected to operate at full capacity until Year 4 and so staffing costs will be lower. All costs grow with inflation.
Operating expenses (Years 4-15)	$4,808,584	
Capital from philanthropy	$0	Conservatively set to $0 because a publicly funded project cannot guarantee the success of philanthropy prior to it being provided.
Photon treatment cost per course	$12,150	Children and adults with cancers of the brain or spinal cord or in close proximity to such structures currently receive 30 or even 35 treatments of standard RT. The estimated cost of an avoided course of standard RT is $12,150, which is the 2015-16 Interprovincial Committee agreed charges for 30 fractions of curative-intent RT, at $405 per fraction.
Depreciation life (years)	15	15 years is a conservative estimate of the lifespan of a PBT unit. A similar UK strategic case assumed a 20-year facility life (UK Department of Health, 2012). Depreciation will be calculated using a straight-line method.

Cost/benefit analysis

A cost/benefit analysis consisting of a quantitative financial analysis from a capital budgeting perspective was conducted. Net present value (NPV), an important output of a capital budget, is the sum of all cash flows over the life of the project expressed in current dollars. The NPV is the impact (costs or savings) of the proposed change compared to the status quo in present value dollars. In the private sector, a positive NPV generally means that a project is approved unless the project requirements exceed available resources; in which case, projects are ranked according to the magnitude of their NPV. In the public sector, projects may have a negative NPV due to the lack of associated revenue. A negative NPV does not necessarily mean that a project should be rejected but rather that the societal benefit must warrant the cost. In this paper, NPV was calculated and its sensitivity to various assumptions tested.

The cost/benefit analysis was performed from the perspective of Alberta public sector financial impacts (hereafter, “the Province”), using a conservative, 15-year time horizon, commencing in 2024. Year 2024 was chosen because it is the year that first patient treatments are scheduled to occur in the new Calgary cancer center currently under construction. Costs, savings, and revenues were estimated based on current dollar values, applied to 2024, and adjusted for inflation at 2% per year for the life of the project [24]. All values are reported in Canadian dollars.

Patient numbers

A compact, single-vault PBT facility in Alberta would provide the capacity to treat over 250 patients per year, with half this volume allocated to out-of-province patients on a cost-recovery basis. Table 2 projects the PBT patient numbers for this proposal. These assumptions limit Albertan patients to those with ‘Category A’ indications [1] for PBT. Note that ocular melanoma patients requiring a low energy proton beam would not be treated at the proposed facility. It was assumed that the PBT facility would not operate at full capacity until year four due to gradual adoption within Alberta and from other provinces.

**Table 2:** Estimated numbers of patients avoiding USA referral for proton beam therapy (PBT) and Albertans and out-of-province patients receiving PBT in Alberta RT: radiation therapy

Year	1	2	3	4	5	10	15	Facility Lifetime Total
Year	2024	2025	2026	2027	2028	2033	2038	
Albertans who would have had PBT in USA*	21	22	22	23	24	27	32	391
Albertans who would have had standard RT but will receive PBT if it were available within the province	39	49	61	79	80	85	90	1165
Patients coming to the centre for PBT from out-of-province*	60	75	94	100	103	119	138	1648
Total patients treated annually with PBT	120	145	177	202	207	232	261	3203

In 2015-16, 15 Albertans were funded to receive PBT in the USA compared to 13 in 2013-14 and five in 2011-12 (private communication, Stella Hoeksema, previous chair, Out-of-Country Health Services Committee). The Ministry of Health's mean cost for 15 patients who received PBT in the USA in 2015/16 was $237,248 per patient. It is estimated that by 2024, if there is no Canadian facility, 21 Albertans per year will be funded to receive PBT in the USA, and the annual number of cases would increase by 3% per year, parallel with rising cancer incidence, to be 32 per year by 2038. A new PBT facility would have the capacity to treat 100-200 patients per year during a three-year start-up phase, 200 per year by Year 4, and increasing by 3% annually thereafter, to a maximum of 261 in 2038. Approximately half of the annual treatment capacity would be reserved for non-Albertans. The indications for PBT or the adoption/implementation of current indications could expand or contract so USA referral rates of 0 to 35/year were considered in the sensitivity analysis.

Positive cash flows

Most public sector projects do not contain a revenue component in the capital budget; however, the PBT capital budget contains two material cost savings and one revenue source (Table 2):

- Alberta currently incurs costs each year to send patients to the USA for PBT. This expenditure would cease if PBT was available in the province. Many patients with indications for PBT are not able or willing to accept out-of-country treatment for social or financial reasons so it was estimated that the number of patients treated with PBT would be substantially larger if PBT was available within the province.

- Many patients receiving PBT in the new facility would have otherwise received standard RT within Alberta if not sent to the USA for PBT. The avoided costs of delivering standard RT are accounted for in the capital budget.

- It was anticipated that 50% of the treatment capacity of the PBT facility would be available for the care of patients from out-of-province with indications for PBT. Other provinces would be charged a cost-recovery fee, estimated at $45,000 per PBT course (based on expert opinion; sensitivity range $30,000-$70,000 per course). This cost recovery would be substantially less than the cost of PBT in the USA.

Negative cash flows

There are typically two major expense components associated with projects: capital expenses and operating expenses.

Capital expenses

There are three components of capital expenses, each estimated with considerable uncertainty, but a total initial investment of $40 million would cover all three capital cost requirements for a single beam line PBT built as part of an existing cancer center construction project. The capital costs would be very much larger if developing a PBT facility on a ‘green-field’ site or if a multi-beam line PBT facility was required.

Equipment

Estimates provided by prospective vendors indicate a single-beam line PBT unit, including the cyclotron or synchrotron, beam-line and treatment delivery device, would cost approximately $30 million.

Facility

Significant ancillary systems are needed to operate a PBT facility, including patient and staff facilities; immobilization and simulation (imaging); treatment planning hardware and software; preventative maintenance; and quality assurance of treatment beam delivery units. In the current analysis, the PBT facility was assumed to be incorporated into a new or existing comprehensive cancer center, which resulted in significant cost savings both for capital and operating costs. For instance, the incremental treatment planning and patient visits of a single PBT beamline to the new Calgary cancer center might increase treatment planning and patient visits to the RT department by just 2% compared to the planned capacity. This volume increase could be accommodated within the planned capacity. Capital costs estimates also included software and hardware for planning, quality assurance, increased shielding requirements, as well as space for these functions. Incremental facility construction costs were estimated at $10 million.

Start-Up

To attract patients from other Canadian provinces, oncologists in other centers must feel confident that treatment quality in the Alberta facility would be equivalent to that in the USA. An initial investment to develop a clinically based research program, treating all patients on prospective clinical trial protocols and strategic recruitments may increase the credibility of a Western Canadian PBT service. The costs of or income from a sustained research program were not included in the model. Ideally, such costs should be offset through funding from philanthropy and granting agencies. The potential benefits of a Canadian PBT facility on staff recruitment and retention were also not considered in the model.

Operating expenses

Operating expenses consisted of wages for staff, medical service costs, a service agreement, consumables, and electricity (Table 3). A vendor-supported service agreement would be required due to the complexity and uniqueness of the equipment.

**Table 3:** Estimated staffing model and operating expenses per year for start-up and full operations

	Years	
	1-3	4-15
	FTE	FTE
Senior/Lead Radiation Therapist (RTT3)	1.0	1.0
Radiation Therapist Level 1 (RTT1)	2.0	4.0
Dosimetrists	1.0	2.0
Medical Physicists	2.0	3.0
Nurses	0.6	1.2
Data Collection / Study Registrar	1.0	1.5
Support	0.3	0.5
Administrative Assistant	0.5	0.5
Clerk	0.25	0.5
Salaries	$ 1,295,883	$ 2,131,040
Medical Services	$ 224,094	$ 448,187
Service Agreement	$ 2,000,000	$ 2,000,000
Electricity	$ 200,000	$ 200,000
Consumables*	$ 19,763	$ 29,357
Total	$ 3,739,739	$ 4,808,584

Costs not included in the model

The justification for PBT was its potential to reduce side effects and increase the probability of cure [14]; however, these benefits were not included in the model. A PBT service would decrease the annual number of Albertan patients receiving standard RT by 0.5%-1%, which would have a minor impact on existing standard RT capacity. However, there is an opportunity cost of not expanding standard RT services. Most of the cost of developing and running a major research program are also not included here.

## Results

Net free cash flow

**Figure 1:**
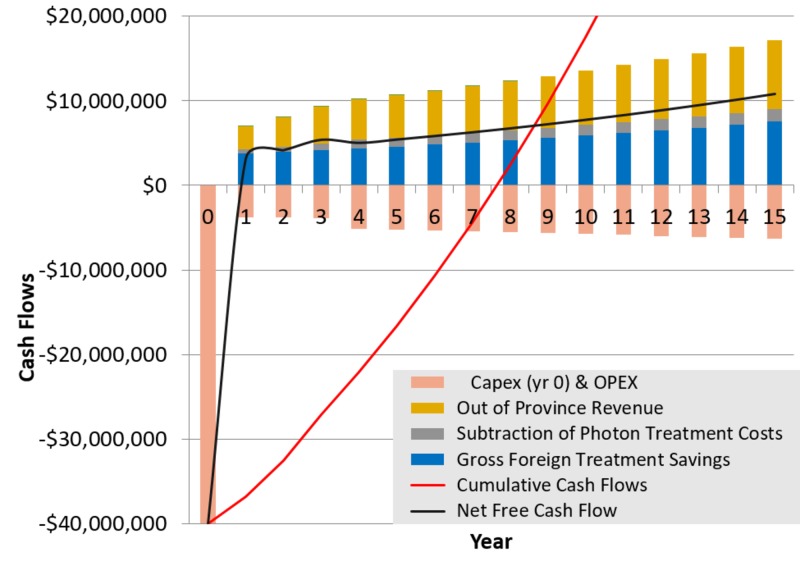
Estimated cash flows for a compact proton beam therapy (PBT) facility in Western Canada A $40 million capital expense is incurred in Year 0 (installation cost), which is recouped by Year 8, after which the cumulative cash flow (similar to net present value (NPV) but uncorrected to current dollar values) is positive (red line). The initial capital expense (CAPEX) and annual operating expenses (OPEX) are shown as negative bars (pink below the $0 line). Positive cash flows include revenues from treating out-of-province patients (yellow), avoided standard radiation therapy courses (grey), and avoided cost of USA referrals ("gross foreign treatment savings in blue) above the $0 line. Overall, the 15-year financial impact of adding a PBT to service to an existing large cancer center construction project in Western Canada is overwhelmingly positive.

After an initial capital outlay of $40 million for PBT equipment and incremental facility costs, the project would achieve a positive cash flow in year one and maintain positive cash flows through the life of the project (Figure 1). In this analysis, the estimated payback period, that is, the estimated time to recoup the initial capital investment, was eight years of operation. With no PBT facility in Western Canada, Alberta would spend an estimated $70.4 million to send 391 patients to the USA for PBT over the 15-year period of this analysis (2024 to 2038, Table 2). The present value (in 2024) of all cash flows associated with this project was a saving of $42.3 million over 15 years, as shown in Table 4. This means that the province would spend $42.3 million less between 2024 and 2038 by building a PBT facility instead of maintaining the status quo of sending patients to the USA for PBT. The positive cash flow was driven by avoided costs of referring Albertans for PBT in the USA and a modest, cost-recovery charge for patients referred to Alberta from other Canadian provinces.

**Table 4:** Financial model for a Western Canadian proton beam therapy facility

Year	0	1	2	3	5	10	15
Year		2024	2025	2026	2028	2033	2038
Estimate of Incremental Gross Savings							
Savings from avoided treatments in the USA		$3,780,000	$3,971,268	$4,172,214	$4,605,125	$5,894,248	$7,544,237
Photon RT costs avoided (Table 2)		$473,850	$604,159	$770,302	$1,052,519	$1,236,846	$1,448,140
Total Incremental Net Savings		$4,253,850	$4,575,427	$4,942,517	$5,657,643	$7,131,093	$8,992,378
Estimate of Initial and Incremental Costs							
Capital Expense	-$40,000,000						
Operating expense: low throughput adjustment during ramp-up		$1,068,845	$1,090,222	$1,112,026	$0	$0	$0
Annual operating expense		-$4,808,584	-$4,904,756	-$5,002,851	-$5,204,966	-$5,746,703	-$6,344,824
Total Costs	-$40,000,000	-$3,739,739	-$3,814,534	-$3,890,824	-$5,204,966	-$5,746,703	-$6,344,824
Out of province cost recovery							
Out of province revenue		$2,700,000	$3,442,500	$4,389,188	$5,017,073	$6,421,514	$8,219,102
Net Free Cash Flow	-$40,000,000	$3,214,111	$4,203,393	$5,440,880	$5,469,750	$7,805,904	$10,866,656
NPV	$42,348,599						

A Western Canadian PBT facility would treat 3203 patients, including 1555 Albertans in the 15-year time horizon of this analysis (Table 2). A PBT facility included in the currently planned Calgary Cancer Project was, therefore, estimated to provide PBT to nearly eight times more Canadians compared to the number of Albertans that would have to travel to the USA for PBT.

Sensitivity analysis

**Figure 2:**
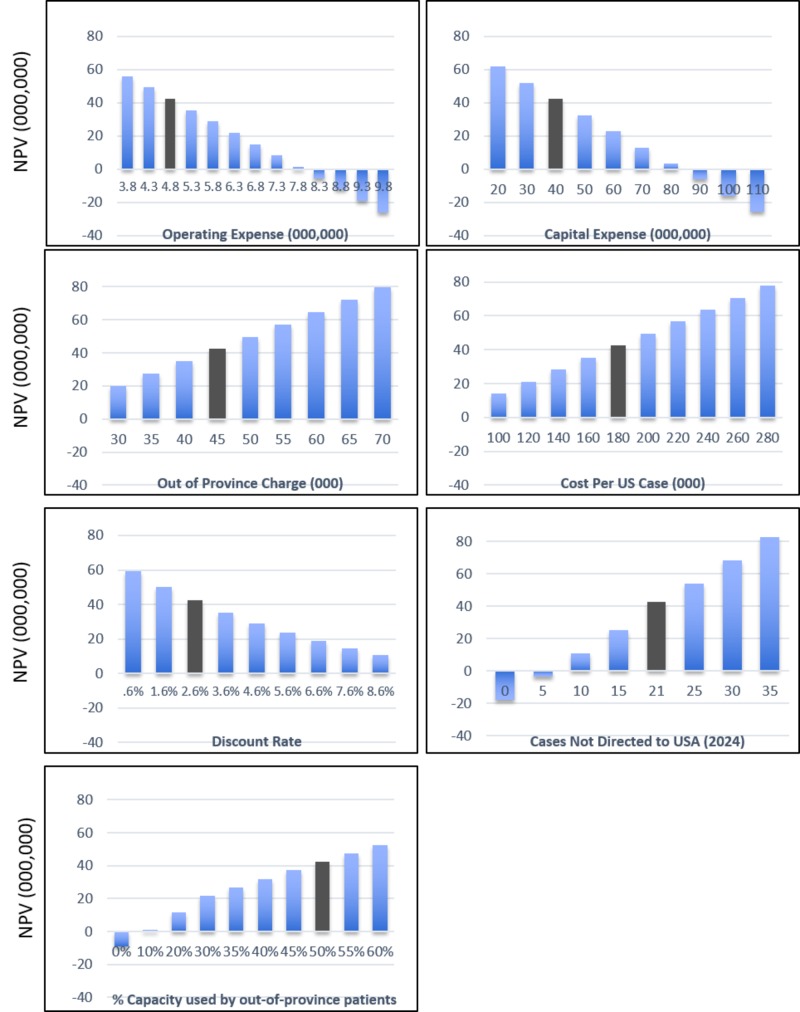
Sensitivity of the financial model to the assumptions Sensitivity of the financial model to the assumptions in this business case is tested by varying the value of each independently. Net present value (NPV) is listed in $ millions. A positive NPV indicates financial viability while a negative NPV must be offset by a societal benefit to justify a project. For financial viability, operating expenses (OPEX) must be capped at $7.5 million per year and capital expenses (CAPEX) below $80 million. The financial model is fairly robust to the other assumptions and remains positive over a reasonable range of values.

The impact of each of the seven main assumptions on NPV was tested by varying each independently (Figure 2). The operating expense per year was estimated (Table 3) at $3.8 million/year in each of the first three years and $4.8 million/year in each subsequent year, growing at the rate of inflation (estimated to be 2% per year) during the facility lifetime. This project would be financially viable unless the annual operating expense exceeded $7.5 million per year in 2016 dollars. Similarly, capital expenses must be less than $80 million.

The financial case was robust to other assumptions, across a reasonable range of values. At least some out-of-province patients (10% capacity) must be funded for treatment for this project to be financially viable. The financial viability of the project was enhanced if the charge for non-Albertans was raised but remained positive even if the cost-recovery charge per patient was reduced to $30,000 per PBT course. A constant out-of-province charge of $45,000 per course of PBT was assumed for the 15-year lifespan of the project, but it could be possible to reduce the charge/course after the capital costs are recovered. With less expensive PBT equipment now available, costs for Canadians sent abroad may decrease; however, NPV remained positive even if the costs of treatment abroad dropped to half of the current charges.

## Discussion

This analysis demonstrated that adding PBT capacity to an existing capital project in Alberta to host the PBT service needs of Western Canadians would be sustainable and could result in significant cost savings for the public health system over the longer-term. Such a facility additionally would expand patient access to PBT with lower overall costs and travel burden for patients and families while achieving improved health outcomes and appropriate care within the region. Currently, patients identified as candidates for PBT are referred to the United States or, if they cannot travel abroad, do not receive PBT and, instead, receive standard RT within their home province. The status quo does not meet an objective of providing appropriate treatment within the patient’s community or region. In many cases, even if PBT is accessed in the USA, it introduces treatment delays and substantial inconvenience for the patient and/or family.

PBT utilization rates in Alberta are considerably lower than the Alberta Health Services guidelines suggest are appropriate [5]. Approximately 85% of Albertans who could benefit from PBT (Category 'A' indications) are not receiving it. There are several reasons for this, including the logistics of arranging out-of-country treatment, border restrictions, complexities of treatment (including surgical and concurrent chemotherapy requirements), and the social, family, and financial resources required to support a patient (especially a child) to receive six or more weeks of PBT in the USA. These practical patient and family logistic issues prevent many patients from accessing PBT even when they meet indications and would receive government funding to access PBT in the USA. Installing a regional PBT facility would create an incentive for intra-provincial and inter-provincial cooperation and collaboration to benefit patients. The service design would be modeled on other regionalized programs, such as pediatric cardiac transplants performed in Edmonton for all of Western Canada [25].

Based on current projections, it is estimated that Alberta will spend $3.78 million treating 21 patients with PBT in the USA in 2024, assuming the mean USA charge per case for PBT drops to $180,000 (from $237,248 in 2015/16). This assumes that clinicians in Alberta do not increase the proportion of patients referred for PBT to meet the conservative 2013 AHS clinical practice guideline indications. Current ‘Category A’ indications [1] suggest that approximately 100 Albertans per year could benefit from PBT. If Alberta were to send even half of Category A patients to the USA, the annual cost would exceed $10 million per year, well in excess of the annual operating costs of the proposed PBT facility that could treat a much larger number of patients within the province. It is estimated that over the first 15 years of clinical operation, Alberta Health would realize a net positive variance of $42.3 million. Under the assumptions in this model, the initial capital investment would be recouped in the eighth year of clinical operations. This is not dissimilar to the estimate made by a recent CADTH evaluation from PBT for all of Canada [26].

The current analysis required that a number of assumptions be made (see Table 1). Realistic estimates were made for all assumptions and the validity of this analysis was tested over a reasonable range of values. For instance, future estimates of patient loads are based on retrospective data. Long-term trends show that the population of Western Canada and the number of cancers diagnosed each year has been steadily increasing since statistics have been kept [22,27]. As such, it is reasonable to believe that future patient loads will be at least equal to linear projections from retrospective data; however, the authors view this as the single most significant risk in this model. Patient projections are similar to, for example, the number of clinically justifiable cases in a recent UK planning study [28]. This analysis also demonstrates the conditions required for the PBT program to be sustainable. Risks include overruns in operating costs, difficulty in developing local expertise in rare tumors, patient reluctance to travel, even within Western Canada, the willingness of other provinces to fund such treatments, and the impact of future facilities elsewhere in Canada.

Philanthropy, which was not taken into account in the current analysis, represents a realistic opportunity to partially or fully offset capital costs. Introducing a PBT service would introduce previously unavailable technology to Western Canada to benefit children and adults with cancer, all causes that, historically, have garnered interest from the community. Having a portion of the capital costs offset would further enhance an already strong fiscal case to introduce PBT within a large, planned cancer center construction project.

## Conclusions

This analysis assessed the financial implications of adding a PBT facility to the current major cancer center construction project in Calgary with a plan to serve the ‘Category A’ indications for PBT for Western Canadians. A Western Canadian PBT facility would provide the clinical benefits of PBT to nearly eight times more Canadians with a net saving to the Alberta government of $42.3 million over 15 years compared to the status quo of funding patients who are willing and able to accept a referral to access PBT in the USA. The costs to implement PBT would be substantially higher and the case for implementing a PBT service, substantially weaker, if a new, free-standing center was required as compared to adding PBT to an existing, large capital project. Similarly, if philanthropy provided some or all of the estimated initial capital investment of $40 million, the business case for proceeding with PBT would be substantially stronger.
